# LurR is a regulator of the central lactate oxidation pathway in sulfate-reducing *Desulfovibrio* species

**DOI:** 10.1371/journal.pone.0214960

**Published:** 2019-04-09

**Authors:** Lara Rajeev, Eric G. Luning, Grant M. Zane, Thomas R. Juba, Alexey E. Kazakov, Pavel S. Novichkov, Judy D. Wall, Aindrila Mukhopadhyay

**Affiliations:** 1 Biological Systems and Engineering Division, Lawrence Berkeley National Laboratory, Berkeley, California, United States of America; 2 Department of Biochemistry, University of Missouri, Columbia, Missouri, United States of America; 3 Environmental Genomics and Systems Biology Division, Lawrence Berkeley National Laboratory, Berkeley, California, United States of America; LSU Health Sciences Center School of Dentistry, UNITED STATES

## Abstract

The central carbon/lactate utilization pathway in the model sulfate-reducing bacterium, *Desulfovibrio vulgaris* Hildenborough, is encoded by the highly conserved operon DVU3025-3033. Our earlier *in vitro* genome-wide study had suggested a network of four two-component system regulators that target this large operon; however, how these four regulators control this operon was not known. Here, we probe the regulation of the lactate utilization operon with mutant strains and DNA-protein binding assays. We show that the LurR response regulator is required for optimal growth and complete lactate utilization, and that it activates the DVU3025-3033 lactate oxidation operon as well as DVU2451, a lactate permease gene, in the presence of lactate. We show by electrophoretic mobility shift assays that LurR binds to three sites in the upstream region of DVU3025, the first gene of the operon. NrfR, a response regulator that is activated under nitrite stress, and LurR share similar binding site motifs and bind the same sites upstream of DVU3025. The DVU3025 promoter also has a binding site motif (Pho box) that is bound by PhoB, a two-component response regulator activated under phosphate limitation. The lactate utilization operon, the regulator LurR, and LurR binding sites are conserved across the order Desulfovibrionales whereas possible modulation of the lactate utilization genes by additional regulators such as NrfR and PhoB appears to be limited to *D*. *vulgaris*.

## Introduction

**Fig 1:**
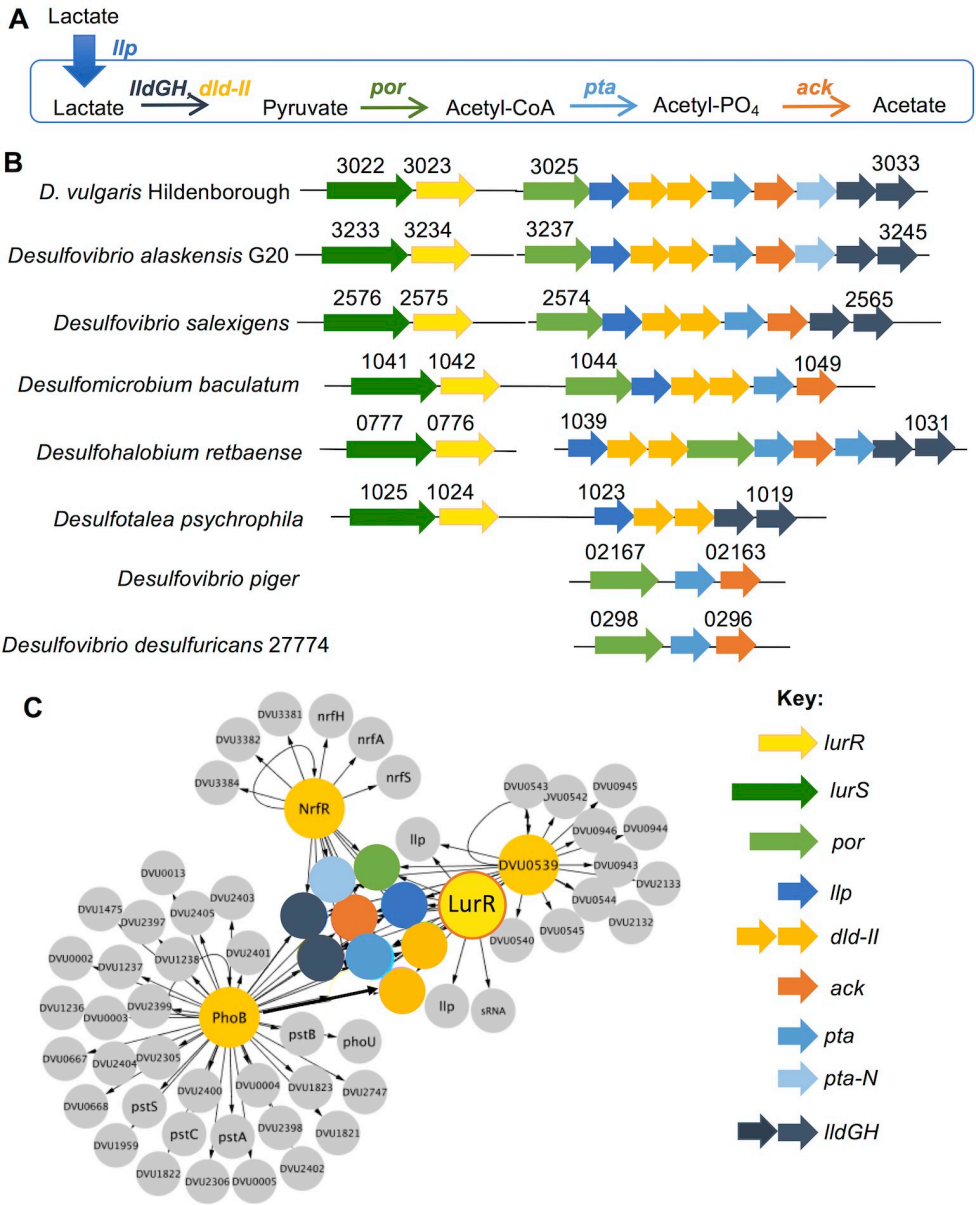
Lactate utilization operon in *Desulfovibrio*. **A**. Lactate is transported inside the cell by a lactate permease (DVU3026, *llp*). D- (DVU3027-3028, *dld-II*) and L-lactate dehydrogenases (DVU3032-3033, *lldGH*) oxidize lactate to pyruvate [6]. Pyruvate is then oxidatively decarboxylated to acetyl-CoA via a pyruvate ferredoxin oxidoreductase (DVU3025, *por*) [7,8]. Acetyl-CoA is then oxidized to acetate in two steps by phosphotransacetylase (*pta*) and acetate kinase (*ack*) enzymes [8]. The operon also contains *pta-N* gene (DVU3031) encoding the N-terminal domain of phosphotransacetylase, whose function is unknown. **B.** The DVU3025-3033 lactate utilization operon and the associated *lurSR* two- component system are conserved across *Desulfovibrio* and related species (also see S1 Table). Gene numbers are indicated above the gene. Genes are color coded according to the key. Gut isolates such as *D*. *piger* have a highly reduced operon and lack *lurSR*. **C.** DAP-chip revealed a regulatory network where four response regulators–LurR, NrfR, PhoB, and DVU0539 –target the DVU3025-3033 genes (colored circles–see key) [9]. Other gene targets are shown in grey circles, and arrows indicate regulatory interactions between an RR and its target. Figure generated using Cytoscape [10].

Sulfate-reducing bacteria such as *Desulfovibrio* play an important role in anaerobic microbial communities in groundwater sediments and reduce toxic heavy metals such as chromium (VI) and uranium (VI) in nuclear waste-contaminated sites [1,2]. These bacteria use organic compounds such as lactate, ethanol, and pyruvate as electron donors for the reduction of sulfate [3]. Addition of lactate compounds to contaminated wells can stimulate growth of the anaerobic microbial community and promote heavy metal reduction [4]. *Desulfovibrio vulgaris* Hildenborough, a model sulfate-reducer, can grow using lactate as the sole carbon source and electron donor (Fig 1A); *D*. *vulgaris* oxidizes lactate to acetate [5]. However, very little is known about the regulation of lactate utilization in these organisms.

The *D*. *vulgaris* lactate utilization operon DVU3025-3033 consists of all the genes encoding the pathway for oxidation of lactate to acetate (Fig 1B). These genes are highly expressed during growth on defined medium containing lactate-sulfate [11] and are conserved across several *Desulfovibrio* genomes and closely related sulfate-reducers (Fig 1B). The operon contains the only copies of the essential *por*, *pta* and *ack* genes; transposon mutant libraries in *D*. *vulgaris* Hildenborough, *D*. *alaskensis* G20 or *D*. *vulgaris* Miyazaki carry no insertions in these three genes [12–14]. However, the lactate permease, D- and L- lactate dehydrogenase genes exist in multiple copies and are not essential. Recently, *D*. *vulgaris* mutants in the *dld-II* and *lldFG* genes were characterized to show that they encode functional D- and L-lactate dehydrogenases respectively [6].

We previously reported a regulatory network that centered on this lactate utilization operon DVU3025-3033 [9]. An *in vitro* DNA Affinity Purification chip (DAP-chip) assay suggested that four response regulators (RRs) directly target the DVU3025-3033 promoter: three sigma54 (σ^54^)-dependent RRs (LurR, NrfR, and DVU0539), and an OmpR family RR, PhoB (Fig 1C). The DVU3025-3033 operon is regulated by both σ^70^- and σ^54^-dependent promoters [6, 11].

LurR, and its predicted cognate sensor kinase, LurS, are encoded directly upstream of the DVU3025-3033 operon. LurR also targets two additional putative lactate permeases (DVU2451 and DVU3284) [9]. Based on *lurSR*’s proximity and its conservation across related genomes, we hypothesized that the LurSR two-component system was likely to serve as the primary regulator for this operon. We also predicted and validated three binding site motifs for LurR (at -389, -350 and -250 bp from the *por* start codon) [9]. NrfR is the response regulator of the nitrite-stress NrfSR two-component system; it activates the nitrite reductase *nrfHA* genes during nitrite stress [15]. PhoB is part of the PhoBR two-component system, predicted to be involved in the phosphate starvation response. A predicted PhoB binding site is located upstream of *por*. The conditions that activate the fourth regulator DVU0539 are unknown. Here, we construct a deletion mutant in the *lurR* gene and analyze the role of LurR on growth, lactate consumption, and expression of the lactate utilization genes. We determine how LurR, NrfR, and PhoB bind the upstream region of DVU3025, and we discuss these findings.

## Results

### LurR is an activator and is required for optimal growth on lactate

**Fig 2:**
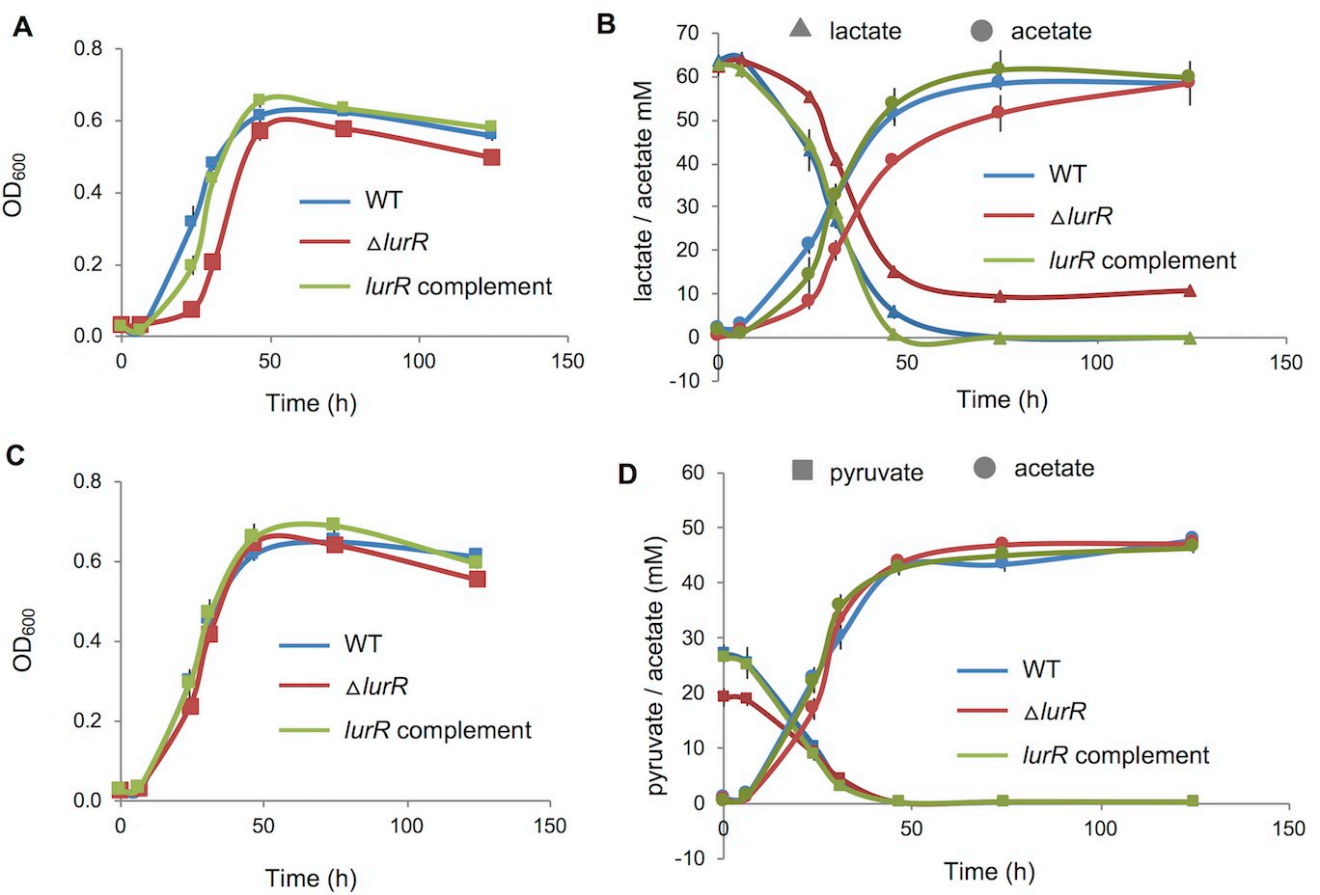
Deletion in *lurR* affects growth and lactate consumption. **A and C**. Growth on lactate-sulfate (**A**) or pyruvate-sulfate (**C**) monitored as OD_600_ readings for WT, Δ*lurR* and Δ*lurR* complemented. Data are average for three biological replicates; error bars indicate standard deviation (For growth curves in log scale, please see S1 Fig). **B and D**. Lactate/pyruvate consumption and acetate production monitored by HPLC during growth on lactate-sulfate (**B**) and pyruvate-sulfate (**D**). Data are average for three biological replicates; error bars indicate standard deviation.

Although a transposon insertion was not obtained in *lurR* during the construction of a transposon mutant library [12], we were successful in constructing a targeted deletion of *lurR* (a bar-coded deletion of DVU3023, BCD1228). We compared the Δ*lurR* mutant with the *D*. *vulgaris* parent strain, JW710 (hereafter called WT), during growth with either lactate or pyruvate as carbon and electron donor, and sulfate as the electron acceptor. The Δ*lurR* strain displayed a longer lag phase during growth on lactate, and lower final cell densities (Fig 2A). The Δ*lurR* showed a lag in lactate consumption that corresponded to its longer lag phase (Fig 2B). The mutant also stopped consuming lactate when the cells entered stationary phase (Fig 2B). Consequently, the mutant produced less acetate and more slowly than WT. The incomplete utilization of lactate could explain the lower final cell densities seen in the mutant. When we complemented the deletion (strain JW9388), the differences in growth lag and lactate consumption disappeared (Fig 2A and 2B). When the strains were grown on pyruvate as carbon and energy source, there were no differences in growth, pyruvate utilization, or acetate production among the three strains (Fig 2C and 2D).

**Fig 3:**
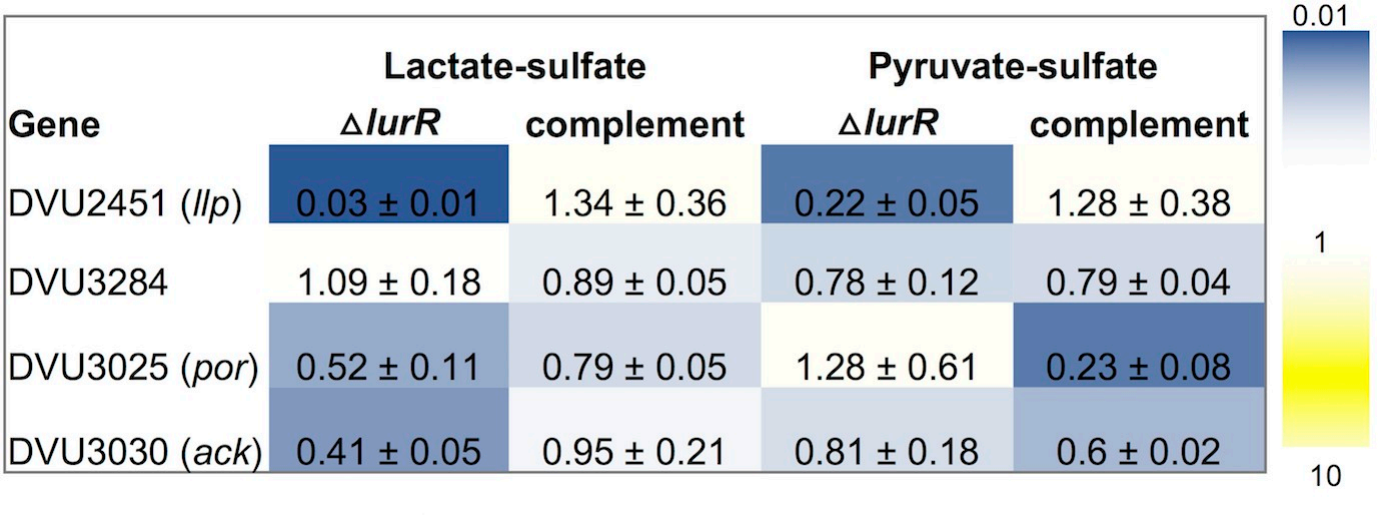
LurR activates lactate utilization genes. Fold changes in expression of select genes were measured by RT-qPCR during growth on lactate-sulfate (LS) or pyruvate-sulfate (PS) of Δ*lurR* and Δ*lurR* complemented strains relative to that of WT. Expression was normalized to that of two reference genes, *rpoD* and *rpoH*. Data are the average from three independent experiments, each with three biological replicates, and error indicates standard deviation.

Next, we examined the expression of *por* and *ack* genes in the Δ*lurR* mutant relative to the wild-type strain by RT-qPCR. Deletion of *lurR* reduced (~ 2-fold) the expression of *por* and *ack*, when the strains were grown on lactate (Fig 3). WT and Δ*lurR* expressed *por* and *ack* similarly when grown on pyruvate (Fig 3). LurR also targets two putative lactate permease genes DVU2451 and DVU3284 [9]. Deletion of *lurR* strongly affected the expression of DVU2451, reducing it by ~33-fold when grown on lactate, and by ~ 4.5-fold when grown on pyruvate (Fig 3). Thus, our data show that LurR activates DVU3025-3033 and DVU2451, and this activation seems to be primarily in response to lactate. When we reintroduced the *lurR* gene into the Δ*lurR* strain (JW9388), expression of *por*, *ack*, and DVU2451 on lactate-sulfate were restored to WT levels. However, for unknown reasons, the complementation decreased the expression of *por* and *ack* genes during growth on pyruvate where the *lurR* deletion itself had no effect. Transcript levels of the second lactate permease gene, DVU3284, were unchanged in Δ*lurR* on both lactate and pyruvate medium.

### Binding of LurR, NrfR and PhoB upstream of *por*

The upstream region of *por* has a predicted σ^54^-dependent promoter (-136 bp from start codon), three binding site motifs for LurR (at -389, -350 and -250 bp from the start codon), and one binding site for PhoB (at -183 bp from the start codon). Previously, we had validated the 16 bp binding site motif for LurR using EMSAs with wild-type and substituted sites [9]. We had also validated the PhoB binding site upstream of the *phoB* gene [9], and the NrfR binding sites upstream of the *nrfHA* genes [15].

**Fig 4:**
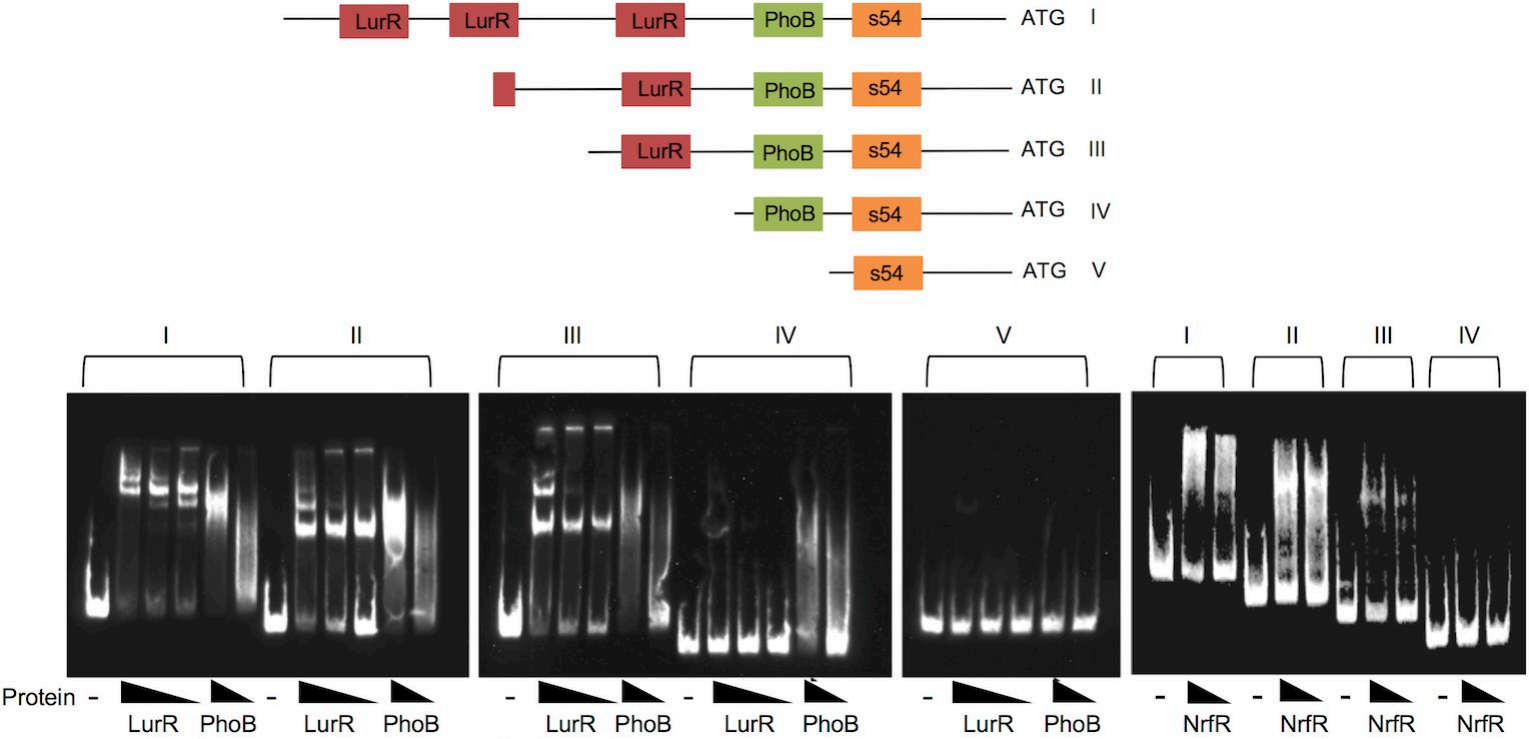
Electrophoretic mobility shift assays with promoter deletions of *por*. The upstream region of the lactate utilization operon contains a σ^54^-dependent promoter (orange box, ATTGGCACATTTCTTGTTA), a predicted binding site for PhoB (green box, AGGTTACAGCATAGTTAC), and three 16 bp binding sites for LurR (red boxes, ATCCGCTTTTTCAGAC, GTCCGCTTTTCAAGAC, and GTCCACTTTTTCAGAC). Five biotin-labeled DNA promoter substrates (I to V) of decreasing lengths (426, 348, 283, 220 and 159 bp) were used in EMSAs with purified His-tagged protein. Protein concentrations used were LurR—2.5, 1, 0.5 pmol; NrfR– 10 and 5 pmol; PhoB– 250 and 125 pmol.

Here, we used truncated *por* promoter substrates containing 0, 1, 1.5 or 3 LurR binding sites in EMSAs. LurR bound all substrates except the promoter fragments (IV and V) lacking any binding motif (Fig 4). NrfR shifted the same fragments as LurR (Fig 4). PhoB shifted all the fragments except for fragment V that does not have a PhoB binding site (Fig 4).

**Fig 5:**
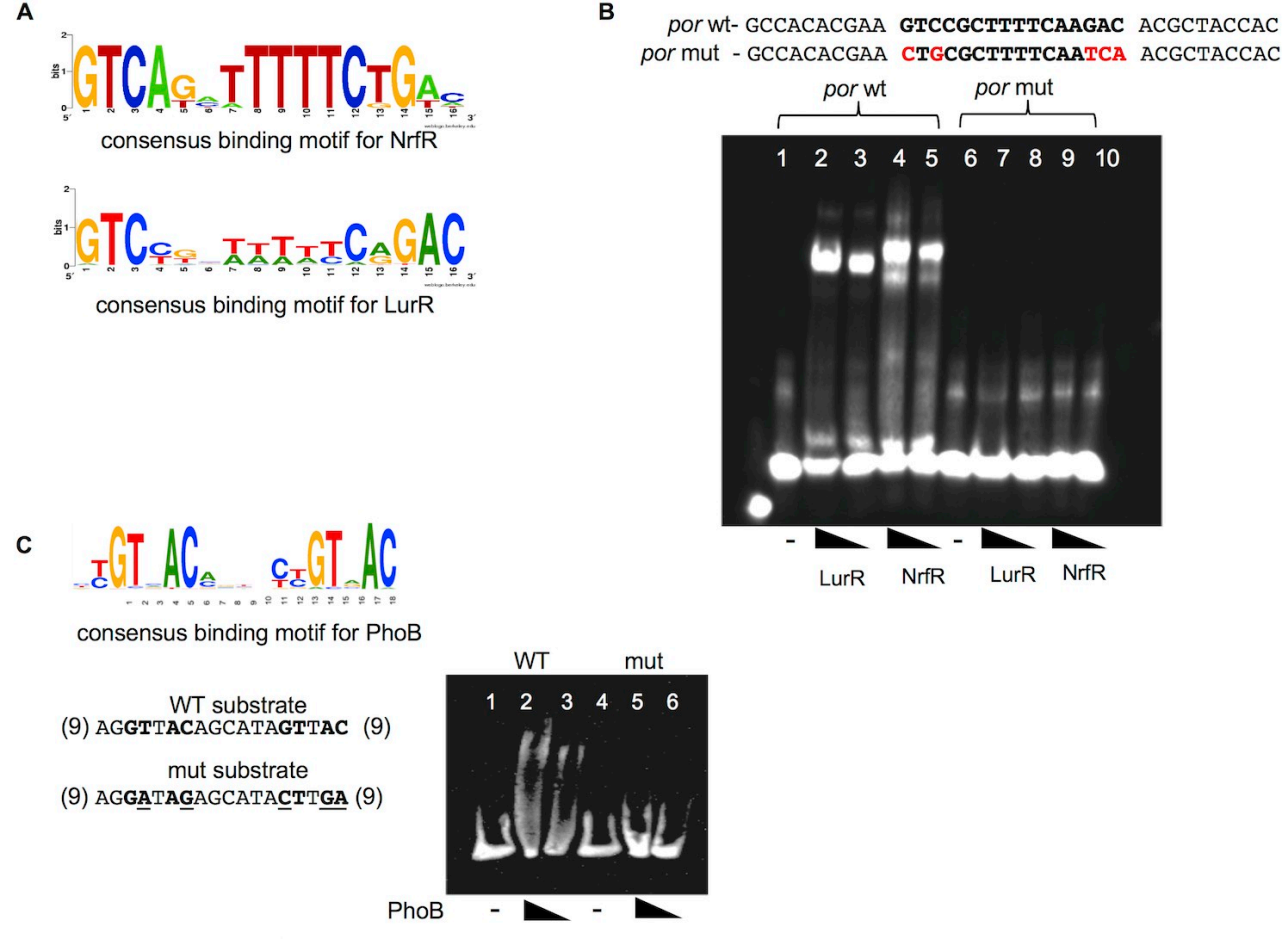
Validation of predicted binding sites. **A.** Comparison of NrfR and LurR binding motifs. Motif images were generated using Weblogo [20]. **B.** NrfR shifts LurR motif. The top strands of the DNA substrates used are shown on top (wt = wild-type; mut = modified). Bases in bold indicate the conserved motif positions, and bases in red indicate the modified bases in the mutated substrate. Lanes 1–5: *por* wt motif; lanes 6–10: *por* mut motif. Lanes 1, 6 –DNA only; lanes 2, 7–1 pmol of LurR; lanes 3, 8–0.5 pmol of LurR; lanes 4, 9–25 pmol of NrfR; and lanes 5, 10–10 pmol of NrfR. **C.** The motif on top indicates the 18 bp consensus PhoB binding sequence [9]. Gel-shift assays with purified His-tagged PhoB protein and the predicted PhoB binding site upstream of *por*. Lanes 1–3: WT substrate (conserved bases are shown in bold); lanes 4–6: mutated substrate (underlined bases indicate substitutions made in the conserved positions); lanes 1 and 4: No protein; lanes 2 and 5: 250 pmol PhoB; lanes 3 and 6: 125 pmol PhoB.

The consensus 16 bp NrfR binding site motif [15] is very similar to the consensus LurR binding motif (Fig 5A). To test if NrfR recognizes the LurR binding site, we performed EMSAs with NrfR and a short substrate containing the LurR binding site found upstream of *por* (Fig 5B). NrfR shifted this substrate. Neither LurR nor NrfR bound an altered substrate with substitutions in the conserved bases, thus indicating that NrfR recognized the same site in the *por* promoter as LurR (Fig 5B). To test if PhoB specifically recognizes the predicted site upstream of *por*, we used a short oligomer DNA substrate containing this predicted PhoB binding site upstream of *por* in an EMSA with purified His-tagged PhoB (Fig 5C). We observed that PhoB shifted the DNA substrate, and this shift was eliminated when substitutions were made to the conserved positions within the motif.

LurR, NrfR and PhoB did not require phosphorylation for *in vitro* DNA binding. Two-component response regulators are activated by phosphorylation by the partner histidine kinase *in vivo*, but *in vitro* DNA binding often occurs without activation [9, 16–19]. Since we lacked purified cognate histidine kinases to phosphorylate the respective RRs, we tested the addition of the small molecule phospho-donor acetyl phosphate in the binding reactions. We did not observe any effect of acetyl-phosphate on DNA binding (not shown). It is also possible that the RRs were purified with some phosphorylation since they were heterologously expressed in *Escherichia coli*. A high amount of PhoB was required for the EMSA reaction, and this may reflect the lack of sufficient activation.

### Effect of NrfR and PhoB on gene expression

We examined *por* gene expression in response to nitrite stress in the wild-type and a *nrfR* transposon insertion mutant by exposing mid-log cultures to 2.5 mM sodium nitrite for 30 minutes [15]. However, we did not observe significant differences in *por* gene expression (not shown).

To observe a physiological effect of PhoB, we first confirmed experimentally using a *phoB* transposon insertion mutant that PhoB activates the high-affinity phosphate transport genes when *D*. *vulgaris* is subjected to phosphate limitation (S2 Fig). However, it proved challenging to assign reliable changes in *por* gene expression by RT-qPCR in a *phoB* mutant. The *phoB* mutant also had a growth defect (S2 Fig) that further hindered collection of adequate cell samples at comparable growth phases.

### Conservation of DVU3025-3033 and *lurSR*

We examined 54 sequenced *Desulfovibrio* genomes available on the IMG (Integrated Microbial Genomes [21]) website, and other closely related species in the orders Desulfovibrionales and Desulfobacterales for the presence of orthologs of *lurR* and the lactate utilization operon (S1 Table). In most *Desulfovibrio* genomes, the *lurSR* genes are encoded proximal to the orthologous DVU3025-3033 operon (S1 Table). The *lurSR* genes are absent in the human microbiome- and rumen-associated *Desulfovibrio* isolates (6 sequenced genomes), and in three environmental *Desulfovibrio* species (*D*. *cuneatus*, *D*. *litoralis*, and *D*. *desulfuricans* DSM 642). Genomes lacking *lurSR* also lacked an organized lactate utilization operon. In the *Desulfovibrionaceae* family, the *lurSR* genes are also present in the five *Pseudodesulfovibrio* genomes, the four *Halodesulfovibrio* genomes, and *Desulfocurvus vexinensis*, but are absent in the human-associated *Bilophila* and *Lawsonia* species (S1 Table). The *lurSR* genes are also represented in the *Desulfomicrobiaceae* (7/7 genomes), *Desulfohalobiaceae* (2/8 genomes) and *Desulfonatronaceae* (5/5 genomes) families. In the order Desulfobacterales, homologs of *lurSR* and the associated lactate utilization operon were found in a few genomes (S1 Table).

We searched for the binding site motif upstream of the first gene in the lactate utilization operons in the other *lurR*-encoding genomes. Most genomes queried had 1 to 4 binding site motifs upstream of the first gene (*por* in most cases, or the lactate permease gene), with the majority of the upstream regions having three binding sites as seen in *D*. *vulgaris* (S1 and S2 Tables).

## Discussion

In this study, we show that the LurR regulator strongly activates the lactate permease gene DVU2451 and moderates the central carbon utilization operon DVU3025-3033 in the model sulfate reducer *D*. *vulgaris* Hildenborough. Activation by LurR depends on the presence of lactate, since deletion of *lurR* affected the expression of the lactate utilization operon during growth on lactate-sulfate, but not on pyruvate-sulfate. The absence of *lurR* also affected the expression of DVU2451 more strongly on lactate-sulfate than on pyruvate-sulfate (~8-fold difference). Even though the expression of DVU3025-3033 was reduced by only 2-fold in the absence of *lurR*, growth and substrate uptake were affected–compared with the WT strain, the *lurR* mutant had a longer lag phase accompanied by a lag in lactate uptake, and lower final cell densities resulting from incomplete lactate utilization. There was no effect on growth or substrate uptake when the strains were grown with pyruvate as the electron donor. Other studies corroborate the key role of LurR in lactate consumption. Transposon insertions in the *lurSR* genes of *D*. *alaskensis* G20 and *D*. *vulgaris* Miyazaki had fitness defects during growth on lactate-sulfate [13, 14]. In the absence of sulfate as an electron acceptor, *D*. *vulgaris* can also grow on lactate in syntrophic association with hydrogen-consuming methanogens. Regulation by LurR may also have a bearing on the upregulation of the lactate utilization operon during coculture with *Methanococcus maripaludis* [22]. High levels of expression of this operon may be reflective of three genes in the operon–*por*, *pta*, and *ack–*being required for growth on pyruvate as well. *D*. *vulgaris* can also utilize molecular hydrogen and formate as electron sources [3], and it is possible that *lurSR* has evolved to express the lactate utilization operon optimally in response to electron and carbon sources. The expression of the lactate utilization operon was altered during several stress conditions such as alkaline stress [23], salt stress [24], salt adaptation [25], air exposure [26], heat shock [27], and peroxide stress [28], where transcriptomics measurements are reported. Since the lactate utilization operon has binding sites for multiple response regulators and two sigma factors, and possibly other transcription factors, the regulation of this operon could be more than a simple activation by LurR alone.

To our knowledge, LurR is both the first two-component response regulator and the first σ^54^-dependent regulator reported to activate lactate consumption. While we have not studied the sensor kinase LurS, domain predictions show that it is a large hybrid kinase with two transmembrane regions, three PAS domains, the histidine kinase domains and a C-terminal receiver domain. Reported regulators that modulate the lactate dehydrogenase and lactate permease genes include FadR family transcription factors in *E*. *coli* [29], *Corynebacterium glutamicum* [30], *Pseudomonas aeruginosa* [31], and *Bacillus subtilis* [32], and LysR-type regulators in *Shewanella* [33] and *Vibrio* species [34]. However, *D*. *vulgaris* has multiple lactate dehydrogenases and permeases [6,35] that likely have their own regulation. For instance, DVU2875 is a FadR family regulator LldR that is predicted to activate the L-lactate dehydrogenase and lactate permease genes in the same operon (DVU2874-3) [36]. The L-lactate dehydrogenase genes DVU1781-83 and DVU2784 are expressed at similar levels to DVU3032-3033. Expression of some of the alternate D- and L-lactate dehydrogenase genes were variably altered in response to deletions of either *dld-II* or *lldFG* [6].

DAP-chip with LurR revealed DVU3284, a third putative lactate permease, also to be a target gene [9]. However, we did not observe changes in expression of this gene under our conditions. This may be due to DVU3284’s role being pertinent in stationary phase growth [37]. The lack of an endogenous control gene in stationary phase made it challenging to test expression changes in this growth phase (the *rpoH* and *rpoD* transcripts used in the exponential phase samples were differentially expressed during stationary phase). The other two permeases DVU3026 and DVU2451 are highly expressed [11], and interestingly, DVU2451 is 88% identical to DVU3026, and in a DVU3026 deletion mutant, DVU2451 expression is increased [6]; thus, they are likely serving redundant roles. The DVU2451 gene is also under the control of a sigma54-dependent promoter [9,11]. LurR has two binding sites at -401 (GTCTGCAATGTCGGAC) and -310 (GTCCATTTTTTCAGAC) upstream of DVU2451 [9].

DAP-chip studies also suggest that three other regulators targeted the lactate utilization operon. Here, we investigated the roles of two of these regulators–NrfR and PhoB. NrfR is also a σ^54^-dependent regulator, and we showed here that NrfR and LurR have very similar consensus binding sites, and that NrfR can bind to the LurR binding motif upstream of *por*. Thus, any regulation of the lactate utilization operon by NrfR likely utilizes the same binding sites as LurR. Although higher concentrations of NrfR were required to shift the *por* upstream DNA as compared with LurR in our *in vitro* EMSAs, it is challenging to extrapolate these observations to *in vivo* protein stoichiometries. In an earlier genome-wide transcriptomics study on nitrite stress in *D*. *vulgaris*, the lactate utilization genes increased by 1.5-2-fold in expression with 2.5 mM nitrite stress added at mid-log and following 4 hours of this exposure [38]. We examined gene expression changes after 30 min of nitrite exposure but did not observe any expression changes in the *por* gene under nitrite stress. Interestingly, few genomes other than *D*. *vulgaris* (including strains DP4 and Miyazaki) encode both *lurSR* and *nrfSR* genes. All the available host gut-associated *Desulfovibrionales* genomes (including *Bilophila* and *Lawsonia* strains) that lack *lurSR* carry the *nrfSR* genes [15]. Thus, possible regulation of lactate utilization by NrfR could be unique to *D*. *vulgaris*.

We confirmed the role of PhoB in responding to phosphate limitation. We showed that PhoB recognizes the predicted Pho box motif present upstream of the *por* gene. The location of the pho box upstream of the promoter region suggests that PhoB would be an activator for this operon (usually transcription factor binding sites for activators occur upstream of the promoter while for repressors occur downstream of the promoter [39]). Since the lactate utilization operon contains the *ack* and *pta* genes, it seems reasonable that the phosphate availability also modulates the operon. The *lurSR* genes of *D*. *alaskensis* G20 play a role in survival after 15 days of phosphate starvation as seen by fitness assays with transposon mutant libraries [40]. However, similar to our observations with NrfR and compounded by the growth defect in the *phoB* mutant, we could not get reliable expression changes of the *por* gene under phosphate-limiting conditions. We were also unable to find PhoB binding motifs upstream of the lactate utilization genes in *lurR*-encoding genomes other than the very similar *D*. *vulgaris* DP4. Therefore, similar to NrfR, any possible regulation of the lactate utilization operon by PhoB may be limited to *D*. *vulgaris*.

Our study highlights the utility of conducting genome-wide assays to query regulator-target interactions. Studying one regulator-target gene interaction at a time may not have revealed the complexity of regulation of critical operons such as the lactate utilization regulon. Our initial genome-wide DAP-chip experiments led us to investigate and discover the cross-talk between the NrfR and LurR regulators. More recently, DAP has been combined with sequencing technologies to enable high-throughput elucidation of regulatory networks [19, 41]. Our work here shows that activation of the lactate utilization operon by LurR is conserved across the *Desulfovibrionales* but fine-tuning of the regulation of this operon in response to other signals/stresses may be unique to each species.

## Materials and methods

### *D*. *vulgaris* growth conditions

*D*. *vulgaris* was grown in defined media containing 8 mM MgCl_2_, 20 mM NH_4_Cl, 2.2 mM K_2_PO_4_, 0.6 mM CaCl_2_, 30 mM Tris, 1 ml/liter of Thauers vitamins [42], 12.5 ml/liter of trace element solution [43], 640 μl/ liter of resazurin (0.1% wt/vol), and supplemented with 50 mM Na_2_SO_4_ and 60 mM sodium lactate (LS4D medium) or 40 mM Na_2_SO_4_ and 60 mM sodium pyruvate (PS4D medium). The pH of the media was adjusted to 7.2 with 1 N HCl. Cultures were grown at 30°C in an anaerobic growth chamber (COY Laboratory Products, Grass Lake, MI, USA) under an atmosphere of 85% N_2_/10% CO_2_/5%. For deletion and transposon mutants, the media were supplemented with the antibiotic G418 (400 μg/ml) (Sigma Aldrich, St. Louis, MO, USA); for JW9388, the complemented Δ*lurR* strain, the medium was supplemented with both G418 (400 μg/ml) and spectinomycin (100 μg/ml).

#### Construction of *D*. *vulgaris* BCD1228 (Δ*lurR*) and JW9388 (Δ*lurR* complemented) strains

The deletion strain BCD1228 (deleted for DVU3023) was constructed similarly as other deletion strains of *D*. *vulgaris* Hildenborough [44]. Briefly, strain JW710 was grown in 20 ml of MOYLS4 [45] overnight. Then, 30 ml of MOYLPS4S3 (30 mM lactate, 30 mM pyruvate, 20 mM sulfate and 10 mM sulfite) was added to the culture and allowed to grow for 3–5 hours. Cells were harvested and transformed by electroporation with pBCD-DVU3023, a plasmid that does not replicate in *D*. *vulgaris* containing a construction that causes a marker exchange deletion of DVU3023 (Δ*DVU3023*::*P*_*KmR*_ *Km*^*r*^*-upp)*. Transformed cells were allowed to recover overnight in MOYLPS4S3 and were then plated in MOYLPS4S3 containing the aminoglycoside G418 (400 μg/ml). Putative transformants were screened for appropriate antibiotic phenotypes (sensitive to 100 μg spectinomycin/ml, confirming the loss of the plasmid sequences, and resistant to G418, confirming retention of *Km*^*r*^). Cell lysates from isolates that passed the initial screen were used as template in a PCR reaction with primers (S3 Table) spanning both sides of the gene targeted for deletion to ensure that the correct double-homologous recombination event occurred (S3 Fig). The expected upstream product of 1057 bp from primers 1 and 2 and the downstream product of 1747 bp from primers 3 and 4 were observed, and these products were sequenced to confirm retention of the wild-type sequences flanking the deleted gene.

The complementation strain JW9388 was constructed by reintroduction of the deleted gene on a plasmid unable to replicate in *D*. *vulgaris*, pMO9380, through antibiotic selection of a double-homologous recombination event. The plasmid was constructed by the Sequence- Ligation-Independent Cloning (SLIC) method [46] with PCR products obtained with the primers (IDT, Coralville, IA) found in S3 Table and the Herculase II DNA polymerase (Stratagene, La Jolla, CA). The plasmid contained DVU3023 and the spectinomycin-resistant gene flanked by the upstream and downstream regions. It was electroporated into strain BCD1228 by previously described methods [45]. Transformed cells were recovered, plated and screened as previously described except that isolates resistant to spectinomycin were selected for further confirmation. Putative complementation isolates were confirmed by Southern blot analysis by digesting genomic DNA with *EagI* (New England Biolabs, Ipswich, MA) and probing with the labeled upstream PCR fragment (Prime-It RmT Random Labeling Kit, Stratagene, La Jolla, CA).

#### Determination of lactate, pyruvate, and acetate

WT, Δ*lurR*, and Δ*lurR* complemented strains were grown on LS4D or PS4D in 40 ml cultures at 30°C. At specific time points, aliquots were withdrawn for OD_600_ measurements on a spectrophotometer, and another 800 μl of the culture was withdrawn and stored at -20°C. For HPLC analysis, the samples were thawed, cells were spun down, and the supernatant was centrifuged through a 10 kDa microspin filter at 14500 x *g* for 5 min. Samples were analyzed by an Agilent 1100 Series HPLC system (Agilent Technologies, Santa Clara, CA) equipped with an Aminex HPX-87H ion-exclusion column (300 x 7.8-mm; Bio-Rad Laboratories Inc., Hercules, CA) 9 μ with guard at 50°C with an injection volume of 5 μl, and a flow rate of 0.6 ml/min with 4 mM H_2_SO_4_ as solvent. Na-lactate, Na-pyruvate, and Na-acetate standards were used to determine unknown concentrations.

#### RNA extraction

Three independent experiments on different days were set up where WT, Δ*lurR*, and Δ*lurR* complemented strains were each grown in triplicate 40-ml cultures of LS4D or PS4D and 1.5 ml aliquot of cells were harvested at mid-log phase (OD_600_ ~0.3), frozen in liquid nitrogen and stored at -80°C. RNA was extracted from the pellets with Agilent Total RNA mini kit following the manufacturer’s protocol (Agilent Technologies, Santa Clara, CA). Genomic DNA contamination was removed by digesting the RNA samples with Turbo DNA-free DNase kit (Life Technologies, Grand Island, NY). The integrity of the RNA was examined visually by agarose gel electrophoresis. RNA was quantified spectrophotometrically on the Nanodrop ND-1000 (Thermo Fisher Scientific, Wilmington, DE).

#### RT-qPCR

500 ng of RNA was reverse transcribed with the iScript RT mix (BioRad, Hercules, CA, USA). The resulting cDNA was diluted ten-fold and 2 μl was used as the template for qPCR reactions. Primers for qPCR reactions were designed using Primer3 (http://bioinfo.ut.ee/primer3/) and are in S3 Table. qPCR reactions were set up in triplicate, with each 20 μl reaction containing 1X SsoAdvanced Universal Sybr Green Supermix (BioRad, Hercules, CA), 500 nM of each primer, and 2 μl of cDNA in 96-well plates (white, BioRad). Reactions were cycled on a CFX96 Real-Time PCR Detection System (BioRad, Hercules, CA) as follows: 98°C/2 min, and 40 cycles of 98°C/10s, 60°C/30 s. RNA integrity was assayed by calculating the relative quantities of the *por* transcript produced by primers amplifying the 3' vs. the 5' end of the gene (ratios close to 1 indicate intact RNA; all ratios were less than 1.2). Expression changes were normalized to that of two σ^70^ reference genes–*rpoH* and *rpoD*.

#### Electrophoretic mobility shift assay

To determine the minimal promoter region of the lactate utilization operon required for binding, DNA substrates were prepared by PCR amplification with a common biotinylated reverse primer and different forward primers (See S3 Table for primer sequences). DNA substrates to test binding site motif predictions were prepared by annealing biotinylated oligonucleotides with their unlabeled complementary strands as described previously [9]. LurR (~57 kDa), NrfR (~56 kDa) and PhoB (~30 kDa) proteins were purified with a C-terminal V5-epitope and a 6X-His tag as described previously [9]. Each protein was mixed with 100 fmol of biotinylated DNA substrate in 10 mM Tris HCl pH 7.5, 50 mM KCl, 5 mM MgCl_2_, and with 1 μg/ml poly dI.dC in a total reaction volume of 20 μl and incubated at room temperature for 20 min. Electrophoresis, blotting and chemiluminescent detection were performed as described previously [9]. Final imaging of the blot was done using the Fluor Chem Q system (Protein Simple, Santa Clara, CA, USA).

## Supporting information

S1 Table Conservation of *lurSR* and the lactate utilization operon across *Desulfovibrio* and related genomes. (XLSX)

S2 Table Conservation of LurR binding motifs. (XLSX)

S3 Table List of strains and primers. (XLSX)

S1 Fig Growth curves from Fig 2 in log scale. (TIFF)

S2 Fig PhoB activates phosphate starvation response. **A.** Transposon insertion in *phoB* results in a growth defect. Growth of WT and *phoB*::mini-Tn5, at 5 and 10% (vol/vol) inoculum sizes on LS4D, monitored using OD_600_ measurements on a Bioscreen C instrument. The growth defect of *phoB*::mini-Tn5 was more pronounced at lower starting cell densities. Data are average of five replicates, and error bars indicate standard deviations. **B.** RT-qPCR measurements of the fold change in expression of the phosphate transport gene *pstS* (DVU2477) normalized to reference gene *rpoH* in WT and *phoB*::mini-Tn5 under phosphate-limiting relative to phosphate-replete conditions. The two strains were grown in LS4D modified to contain 0.1 mM K_2_PO_4_ and 2 mM KCl until mid-log phase. The cells were spun down gently (3000 x g for10 min), and resuspended either in LS4D with 2 mM KCl and 0 mM K_2_PO_4_ (phosphate-limiting) or with 2 mM K_2_PO_4_ (phosphate-replete) and allowed to grow for 1.5 h. Error bars represent the range of fold change as calculated using the standard deviations in the ΔΔC_T_ values (*n* = 3).
(TIFF)

S3 Fig Barcoded deletion of DVU3023 (strain BCD1228). BC1 and BC2 indicate the two barcodes. The grey bars indicate the upstream and downstream homology regions. The DVU3023 gene has been replaced by the kanamycin resistance gene (KmR) and the *upp* gene. Primers 1 and 4 are unique to this construct, outside of the homology regions. Primers 2 and 3 are common to all constructs.
(TIFF)
