# Supplementary figures and images for: LurR is a regulator of the central lactate oxidation pathway in sulfate-reducing Desulfovibrio species
Source: PLoS One. 2019 Apr 9;14(4):e0214960. doi: 10.1371/journal.pone.0214960 (PMC6456213; doi:10.1371/journal.pone.0214960)

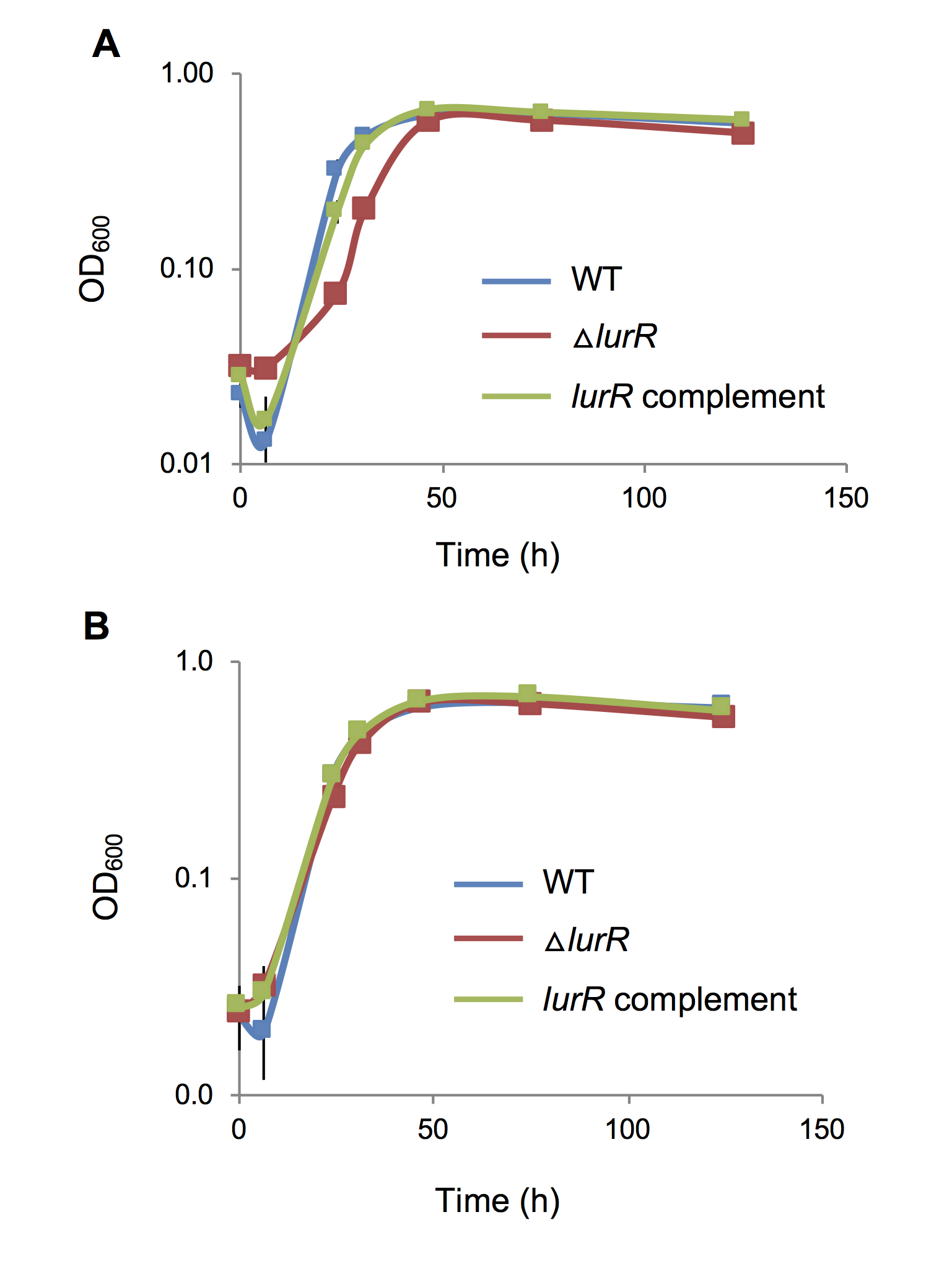

Supplement: S1 Fig — (TIFF) [file pone.0214960.s004.tiff]

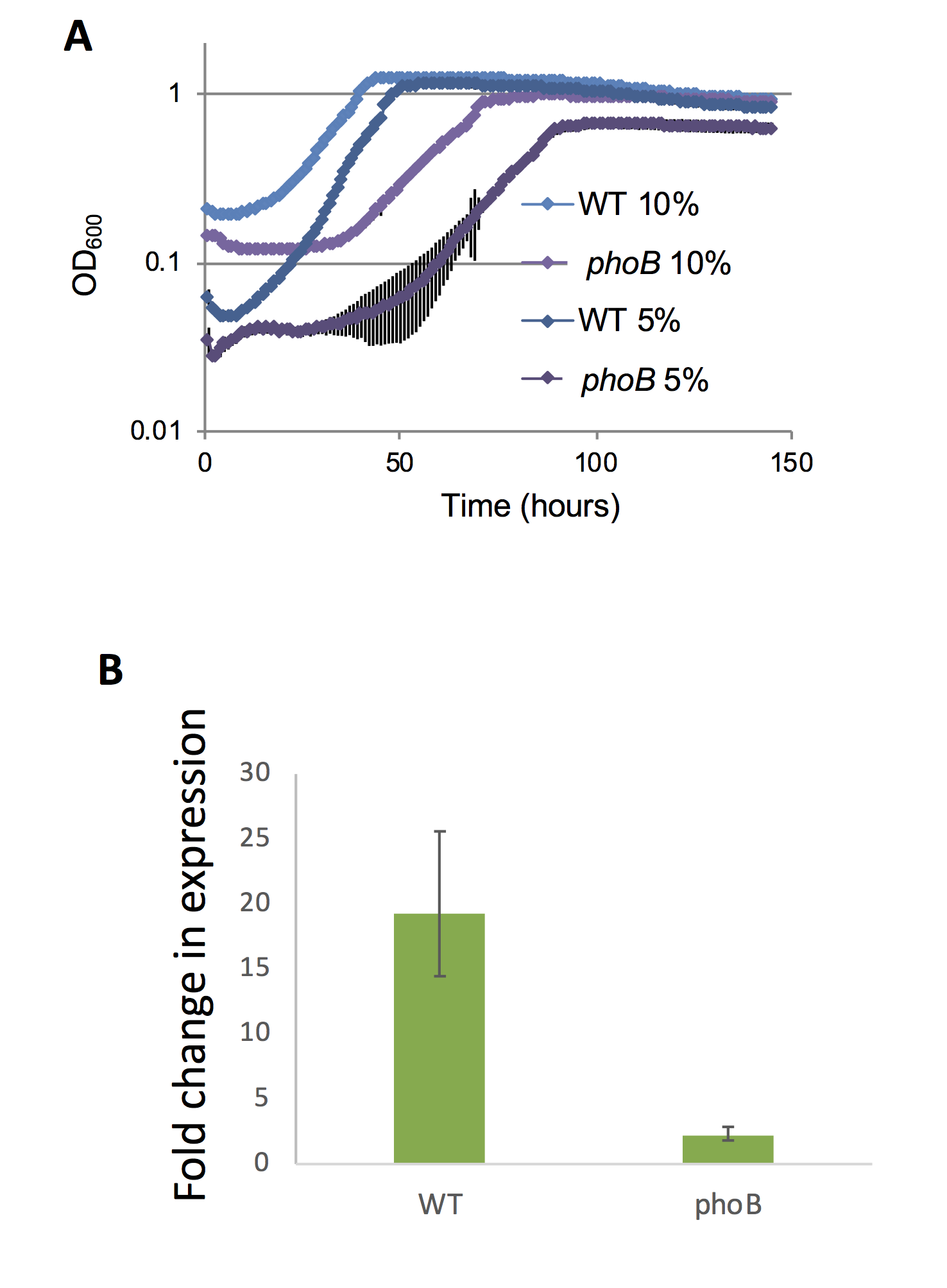

Supplement: S2 Fig — A. Transposon insertion in phoB results in a growth defect. Growth of WT and phoB::mini-Tn5, at 5 and 10% (vol/vol) inoculum sizes on LS4D, monitored using OD600 measurements on a Bioscreen C instrument. The growth defect of phoB::mini-Tn5 was more pronounced at lower starting cell densities. Data are average of five replicates, and error bars indicate standard deviations. B. RT-qPCR measurements of the fold change in expression of the phosphate transport gene pstS (DVU2477) normalized to reference gene rpoH in WT and phoB::mini-Tn5 under phosphate-limiting relative to phosphate-replete conditions. The two strains were grown in LS4D modified to contain 0.1 mM K2PO4 and 2 mM KCl until mid-log phase. The cells were spun down gently (3000 x g for10 min), and resuspended either in LS4D with 2 mM KCl and 0 mM K2PO4 (phosphate-limiting) or with 2 mM K2PO4 (phosphate-replete) and allowed to grow for 1.5 h. Error bars represent the range of fold change as calculated using the standard deviations in the ΔΔCT values (n = 3). (TIFF) [file pone.0214960.s005.tiff]

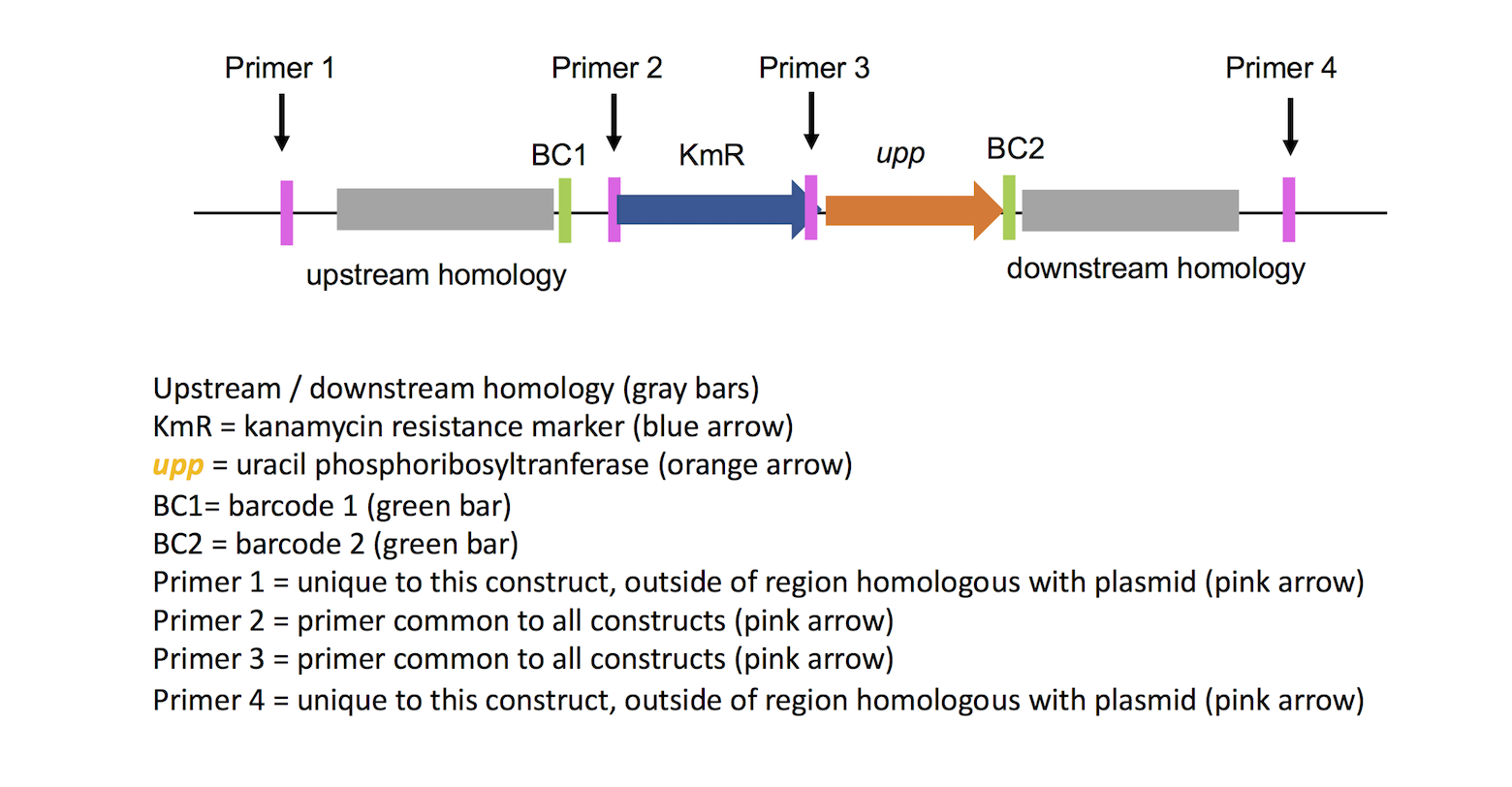

Supplement: S3 Fig — BC1 and BC2 indicate the two barcodes. The grey bars indicate the upstream and downstream homology regions. The DVU3023 gene has been replaced by the kanamycin resistance gene (KmR) and the upp gene. Primers 1 and 4 are unique to this construct, outside of the homology regions. Primers 2 and 3 are common to all constructs. (TIFF) [file pone.0214960.s006.tiff]
